# Effectiveness of Digital Diabetes Management Technology on Blood Glucose in Patients With Type 2 Diabetes at Home: Systematic Review and Meta-Analysis

**DOI:** 10.2196/66441

**Published:** 2025-03-03

**Authors:** Yuping Xiao, Zhenzhen Wang, Lintao Zhang, Nina Xie, Fangyao Chen, Zihao Song, Sha Zhao

**Affiliations:** 1 School of Nursing Shaanxi University of Chinese Medicine Xianyang China; 2 Acupuncture and Moxibustion Department Affiliated Hospital of Shaanxi University of Chinese Medicine Xianyang China; 3 School of Public Health Xi'an Jiaotong University Xi'an China; 4 Department of Clinical Medicine of Traditional Chinese and Western Medicine First School of Clinical Medicine Shaanxi University of Chinese Medicine Xianyang China; 5 Xiangya School of Nursing Central South University Changsha China

**Keywords:** digital diabetes management, type 2 diabetes, patients, home monitoring, blood glucose management, meta-analysis, PRISMA

## Abstract

**Background:**

Patients with type 2 diabetes mellitus (T2DM) face elevated morbidity, mortality, and care costs. Digital self-monitoring of blood glucose (SMBG) can automatically upload data to apps, share the data with health care providers, reduce errors, and aid long-term diabetes management.

**Objective:**

This study aimed to assess the effectiveness of digital diabetes management techniques based on digital SMBG on blood glucose in patients with T2DM at home.

**Methods:**

A systematic search was conducted in PubMed, Embase, Web of Science, China National Knowledge Infrastructure, Wanfang, China Biomedical Literature Database, and Cochrane Library for articles published from the establishment of each database to December 25, 2023. Data were extracted independently by 2 researchers (YX and NX), and the risk of bias in individual trials was rated using the Cochrane risk-of-bias tool. A meta-analysis was conducted using RevMan 5.3 (Cochrane).

**Results:**

Twelve studies were included, involving 1669 participants. The meta-analysis found that in the digital diabetes management group, hemoglobin A_1c_ (mean difference [MD] –0.52%, 95% CI –0.63% to –0.42%; *P*<.001), fasting blood sugar (MD –0.42, 95% CI –0.65 to –0.19 mmol/L; *P*<.001), 2-hour postprandial blood sugar (MD –0.64, 95% CI –0.97 to –0.32 mmol/L; *P*<.001), and BMI (MD –1.55, 95% CI –2.92 to –0.17 kg/m^2^; *P*=.03) were each improved compared to the control group.

**Conclusions:**

Digital diabetes management has been shown to effectively improve blood glucose levels and BMI in individuals with T2DM in home settings. A key feature of successful digital health interventions is the frequent SMBG by patients, supported by dedicated health care professionals who provide timely, personalized, and responsive guidance.

**Trial Registration:**

PROSPERO CRD42024560431; https://tinyurl.com/yfam3nms

## Introduction

Type 2 diabetes mellitus (T2DM) is a chronic metabolic disorder primarily characterized by persistent hyperglycemia due to insulin resistance or inadequate insulin secretion [1]. The International Diabetes Federation [2] reported that the global prevalence of diabetes reached 533 million cases in 2022, with projections indicating a surge to 783 million by 2045, of which over 90% are expected to be T2DM cases [3]. This condition often manifests insidiously, progressing over an extended period, with chronic hyperglycemia as its hallmark clinical feature [4]. Poorly controlled blood glucose levels over time can inflict severe damage on vital organs and tissues, including the heart, brain, and kidneys [5], leading to debilitating complications that significantly compromise patients’ health and quality of life [6].

T2DM is a lifelong condition, and short-term treatment is insufficient to achieve effective blood sugar control. Patients require lifelong management and a long-term, standardized approach to home care [7,8]. International scholars universally agree on the importance of home management for individuals with T2DM [9]. Research by Yan et al [10] has highlighted the high demand among patients with diabetes for home blood glucose monitoring, a finding consistent with the observations by Simsek et al [11] regarding chronic disease management in older adult populations. An increasing number of patients now seek personalized, home-based guidance from health care professionals (HCPs), underscoring the pivotal role of the home environment in diabetes rehabilitation [12,13]. Moreover, studies demonstrate that home blood glucose self-monitoring effectively improves glycemic control and reduces the risk of long-term complications [14].

Traditionally, home blood glucose management has relied on clinician-led, face-to-face consultations. However, such offline consultations are time-consuming for HCPs and impose economic burdens on patients. In addition, these short-term interactions often fail to adequately support patients’ long-term self-management efforts, rendering them less effective [15].

Mobile internet technology has brought about a revolution in diabetes management by introducing digital platforms designed for home-based patients with T2DM. Digital diabetes management entails using advanced tools to gather self-monitoring of blood glucose (SMBG) data, which is subsequently sent to HCPs for analysis and feedback. This technology simplifies blood glucose monitoring and boosts patient-provider communication [16]. Research indicates that it allows HCPs to assess data in real time, reducing the necessity for outpatient visits and easing the burden on both health care facilities and patients [17].

Despite its benefits, the manual entry of SMBG data during home care remains time-intensive, particularly for older adult patients, and is prone to errors, including transcription inaccuracies or intentional misreporting [18,19]. In addition, HCPs often face challenges in interpreting SMBG data to identify lifestyle or treatment issues. Recent advancements in SMBG devices now allow automatic uploads of blood glucose measurements to secure digital health applications accessible to HCPs [20-22]. Real-time monitoring of this data enables timely interventions when abnormal readings are detected, thereby facilitating more accurate management of hypoglycemia or hyperglycemia. Furthermore, this technology supports long-term diabetes care by informing adjustments to insulin, diet, and exercise regimens [23].

Currently, no meta-analysis has systematically assessed the effectiveness of digital SMBG-based diabetes management technologies. Evaluating the impact of these technologies on blood glucose control in home settings is essential for understanding their utility in managing T2DM. This study aims to synthesize global research findings through meta-analysis, examining the effects of digital SMBG-based diabetes management on glycemic control in home-based patients with T2DM. The results are intended to provide evidence to support the broader application of digital SMBG technologies in home care environments.

## Methods

### Overview

This review followed the PRISMA (Preferred Reporting Items for Systematic Reviews and Meta-Analyses) guidelines published in 2020 [24], and a search protocol retrospectively registered on July 5, 2024 on PROSPERO (CRD42024560431) formed the basis of the review process.

### Search Strategy

We searched PubMed, Web of Science, Embase, Cochrane Library, China National Knowledge Infrastructure, Wanfang, and the Chinese Biomedical Literature Database using the terms “Diabetes Mellitus, Type 2,” “Internet,” and “RCT” from inception until December 25, 2023. The search strategy used Boolean logic to combine medical subject headings and text word searches. We also screened references from relevant previous systematic reviews as additional sources, and the languages were limited to Chinese and English. The complete search strategy can be found in Multimedia Appendix 1.

### Eligibility Criteria

Inclusion criteria were formulated based on the populations, interventions, comparisons, outcomes, and study design (PICOS) framework:

1. Populations: Participants with T2DM
2. Interventions: Patients used a digital blood glucose management system that allowed for the automatic upload of SMBG data from home to a secure platform via Bluetooth or other wireless technologies, and HCPs provided timely and responsive personalized guidance
3. Comparisons: Traditional home blood glucose monitoring methods (including routine outpatient follow-up, blood glucose monitoring logs, etc)
4. Outcomes: Hemoglobin A_1c_ (HbA_1c_), fasting blood glucose (FBG), 2-hour postprandial blood glucose (PBG), and BMI. HbA_1c_ is the gold standard for long-term blood glucose control, reflecting the average blood glucose levels over the past 2 to 3 months. FBG can present information on blood glucose in the fasting state, which helps to understand basic glucose metabolism. Measuring the PBG can evaluate the body’s ability to handle the glucose load after a meal and provide insights into the PBG regulation. BMI is a practical tool for assessing overall body fat, which is associated with health risks caused by obesity and metabolic disorders, and the included studies reported at least one of these findings.
5. Study design: Randomized controlled trials

Exclusion criteria included (1) studies involving patients with gestational diabetes or type 1 diabetes, (2) review or discussion papers, and (3) studies with insufficient data.

### Study Selection

Two reviewers (YX and NX) independently performed the screening and selection of studies. Disagreements were resolved through discussion or consultation with a third reviewer (ZW). First, we imported the retrieved literature into the NoteExpress software (Beijing Aiqinhai Software Company) and used it to identify and remove duplicate literature. Then, we evaluated whether the titles and abstracts were eligible. In addition, clearly incompatible studies were excluded after screening the titles and abstracts. Finally, full texts of qualified studies that strictly adhered to the inclusion and exclusion criteria [25] were screened to determine their final eligibility for the review.

### Data Extraction

Data extraction from the studies included was performed by 2 independent reviewers. Characteristics of the studies (author, year, country, and sample size) and intervention characteristics (equipment used by the intervention, intervening measures, control measures, duration, and outcomes) were included.

### Risk-of-Bias Assessment

The quality of the included studies was assessed using the Cochrane risk-of-bias tool, administered by 2 independent assessors. This tool evaluates the quality of study methodology across seven core dimensions: (1) the process of randomization sequence generation, (2) the concealment of allocation, (3) blinding of participants and researchers, (4) blinding during outcome assessment, (5) completeness of outcome data and handling of missing data, (6) selective reporting of study results, and (7) the potential for other sources of bias. The risk of bias for each domain was categorized into 3 levels: low, unclear, or high. Disagreements between the assessors were resolved through discussion, or, if necessary, consultation with a senior reviewer (ZW).

### Data Analysis

The meta-analysis was conducted using RevMan 5.3 [26], a desktop version of Review Manager software used for Cochrane intervention and flexible reviews. For continuous outcomes, we calculated the mean difference (MD) as a metric; while for dichotomous variables, the risk ratio was used and visualized by forest plots. In each analysis, *I*^2^ was used to measure statistical heterogeneity among studies. According to the values of *P* and *I*^2^, the fixed-effect model (*P*>.10, *I*^2^<50%) or random-effects model (0<*P*<.10, *I*^2^≥50%) were selected [27].

## Results

### Results of Literature Search

**Figure 1:**
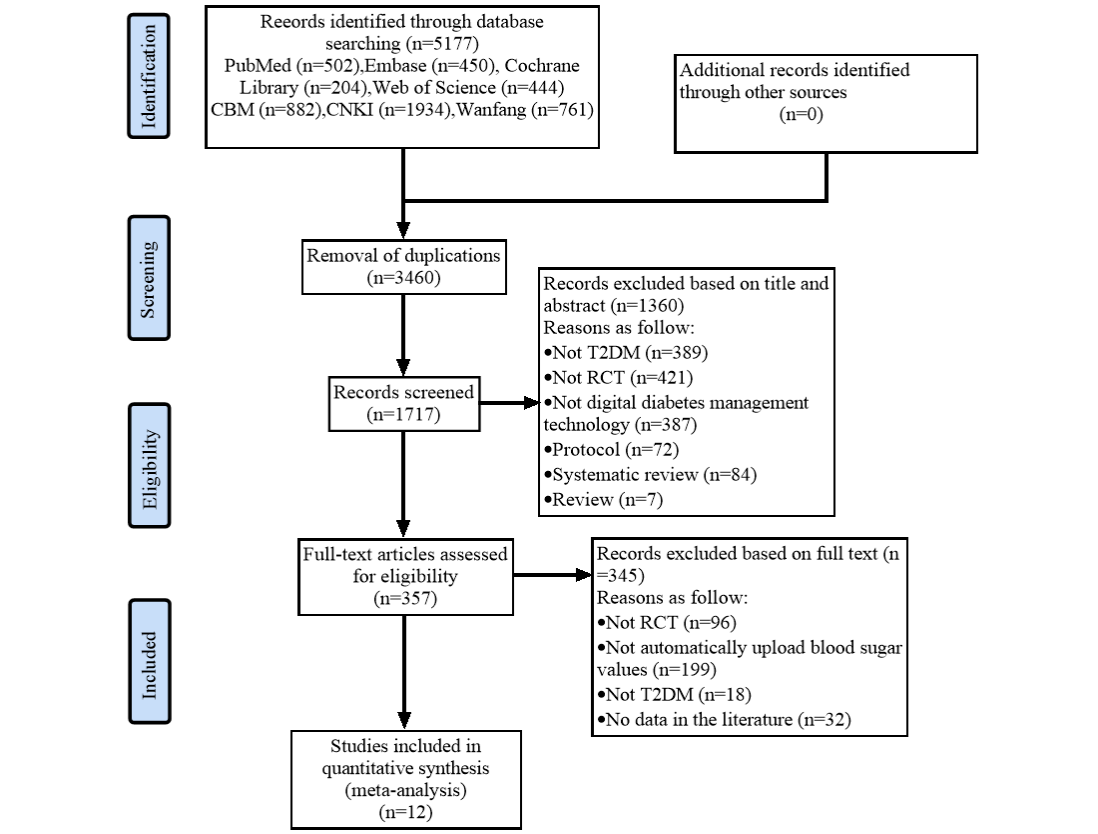
PRISMA (Preferred Reporting Items for Systematic Reviews and Meta-Analyses) flow diagram: papers included and excluded in this review. RCT: randomized controlled trial; T2DM: type 2 diabetes mellitus.

A total of 5177 papers were retrieved from 7 databases. After removing 3460 duplicate records, 1360 articles were deleted due to unqualified titles and abstracts. A more detailed full-text screening was conducted on the remaining 357 articles, and 345 of them were excluded for not meeting the established criteria. As a result, the final systematic review included 12 meta-analysis studies [28-39]. The detailed research screening process and results are shown in Figure 1.

### Study Characteristics

The research includes trials conducted in 5 countries: China [28-34,36], the United States [35], India [37], the United Kingdom [38], and Italy [39]. These trials were published between 2015 and 2023. The sample size ranged from 64 to 285 participants, and the intervention duration varied from 1 to 12 months. Among the 12 studies, 6 used Bluetooth technology to achieve automatic upload of blood glucose values [28,29,32,34,38,39], one study adopted an implantable blood glucose sensor for automatic data transmission [33], and another 5 studies used local area network technology to automatically upload blood glucose measurement values [30,31,35-37]. Multimedia Appendix 2 [28-39] presents the author, year, country, sample size, equipment used by the intervention, intervening measure, control measure, duration, and outcomes.

### Risk of Bias

**Figure 2:**
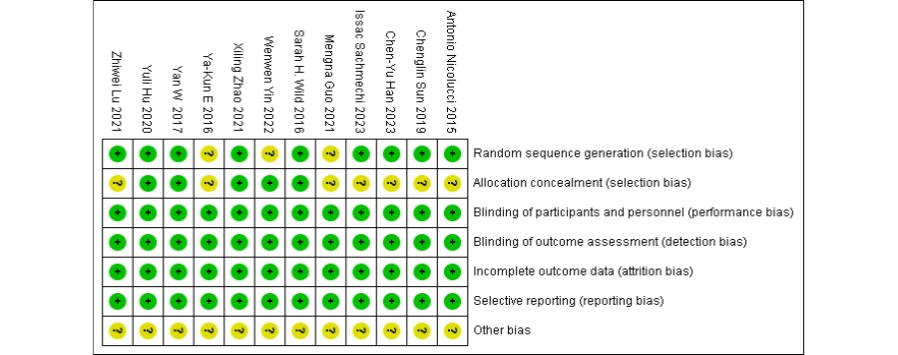
Risk-of-bias analysis of each included study.

**Figure 3:**
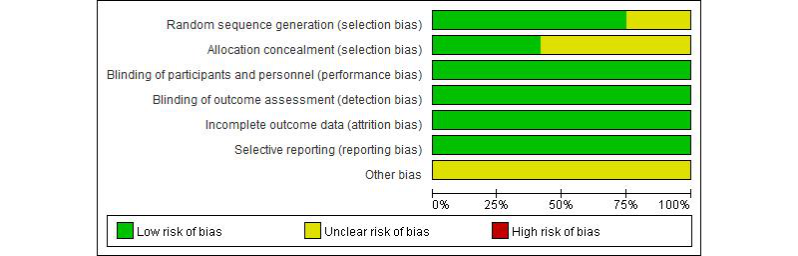
Overall risk-of-bias analysis of included studies.

Based on the Cochrane criteria, a risk-of-bias assessment is presented in Figures 2 and 3 [28-39]. A total of 12 studies were included, all of which were assigned a literature quality grade of B [28-39]. Randomized methods were reported in detail in 9 (75%) of the 12 studies [28-30,34-39]. The remaining 3 studies did not provide detailed randomization methods. In terms of allocation concealment, 5 studies explicitly mentioned the methods used [30,32,35-37]. In all studies, participants were not blinded due to the specificity of the intervention method.

### Results of the Meta-Analysis

#### Effects on HbA_1c_

**Figure 4:**
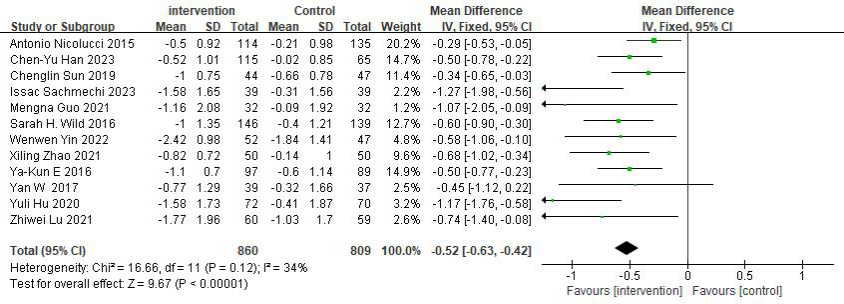
Forest plot of hemoglobin A_1c_ (HbA_1c_).

Twelve studies reported the effectiveness of digital diabetes management in improving HbA_1c_ [28-39]. The meta-analysis results showed a significant improvement in HbA_1c_ (n=1669; MD −0.52%, 95% CI −0.63 to −0.42; *z* value 9.67, *P*<.001; *I*²=34%; fixed-effects model) compared to the control group (refer to Figure 4 [28-39]).

#### Effects on FBG

**Figure 5:**
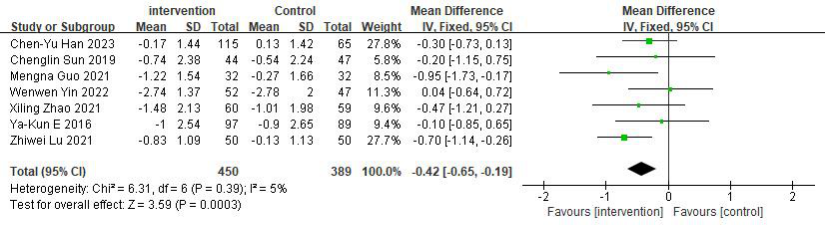
Forest plot of fasting blood sugar.

Seven studies were reported on FBG [28-34]. The results indicated that participants in the intervention group experienced a significantly greater reduction in FBG compared to the control group (n=839; MD −0.42, 95% CI −0.65 to −0.19 mmol/L; *z* value 3.59; *P*<.001; *I*²=5%; fixed-effects model; refer to Figure 5 [28-34]).

#### Effects on PBG

**Figure 6:**
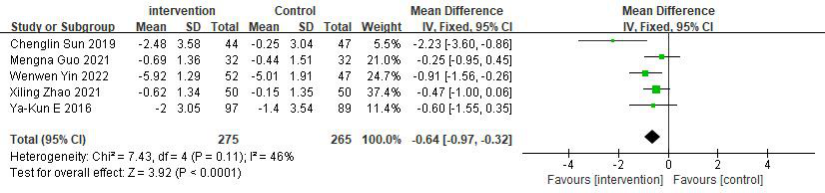
Forest plot of 2-hour postprandial blood glucose.

Five studies reported on PBG [29-33]. The results showed that participants in the intervention group had a significantly greater reduction in PBG compared to the control group (n=540; MD −0.64, 95% CI −0.97 to −0.32 mmol/L; *z* value=3.92; *P*<.001; *I*²=46%; fixed-effects model; refer to Figure 6 [29-33]).

#### Effects on BMI

**Figure 7:**
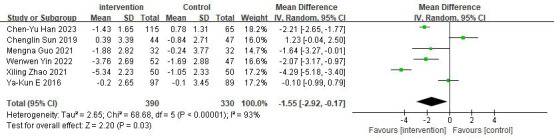
Forest plot of BMI.

Six studies reported on the effectiveness of digital diabetes management in improving BMI [28-33]. The meta-analysis results showed a significant improvement in BMI (n=720; MD −1.55, 95% CI −2.92 to −0.17 kg/m^2^; *z* value=2.20; *P*=.03, *I*²= 93%; random-effects model) compared to the control group (refer to Figure 7 [28-33]). Due to high heterogeneity, 3 studies were identified as contributing to the variability, as revealed by sensitivity analysis [29-31]. After removing these 3 studies, the heterogeneity was reduced to 0% (MD −2.16, 95% CI −2.55 to −1.76 kg/m^2^; *z* value=10.70; *P*<.001).

### Publication Bias and Sensitivity Analyses

**Figure 8:**
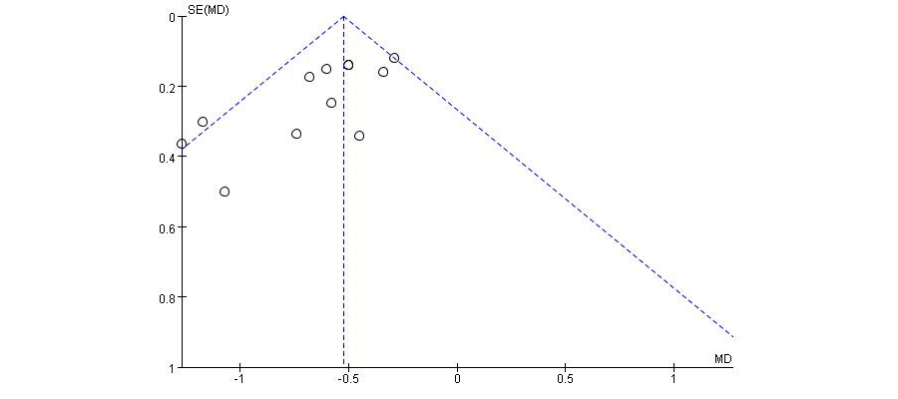
Funnel plot. MD: mean difference.

Most of the points in the distribution of HbA_1c_, an outcome measure, were clustered within the funnel plot; however, the distribution was not entirely symmetrical, suggesting the possibility of publication bias in the results for HbA_1c_ in digital diabetes management (refer to Figure 8). We also performed a sensitivity analysis to assess how the results varied when individual studies were excluded and to evaluate the stability of the findings. When we removed a study from the model and recalculated the pooled estimates for the remaining studies, heterogeneity either disappeared or decreased, and the pooled results remained consistent, indicating the robustness of our findings.

### Subgroup Analysis

Subgroup analysis of BMI based on the length of the intervention revealed that digital diabetes management for 3 months or less resulted in more significant improvements in BMI (refer to Table 1).

**Table 1:** Subgroup analysis.

Outcomes	Studies, n	Participants, n	Heterogeneity		Effect model	Meta-Analysis	
			*P* value	*I*^2^ value (%)		Effect size (95% CI)	*P* value
BMI	6	720	<.001	93	Random	–1.55 (–2.92 to –0.17)	.03
≤3 months	2	164	.005	87	Random	–3.06 (–5.65 to –0.47)	.02
6 months	4	556	<.001	92	Random	–0.84 (–2.37 to 0.69)	.28

## Discussion

### Principal Findings

This meta-analysis evaluated the effectiveness of digital diabetes management technology based on digital SMBG for home-based patients with T2DM. The findings demonstrated significant improvements in HbA_1c_, FBG, PBG, and BMI. Key features of effective digital health interventions include frequent patient engagement in SMBG and timely, personalized guidance provided by specialized HCPs.

Studies have shown that while the compliance rate for blood glucose control among patients with T2DM during hospitalization can reach as high as 80%, this rate significantly decreases by 25% to 30% once they are discharged and transition to home self-management [40,41]. Consequently, there is an urgent need to support home-based patients with T2DM in managing blood glucose fluctuations [42]. This study demonstrates that compared with traditional home blood glucose management, digital diabetes management technologies can significantly improve blood glucose levels in home-based patients with T2DM. Twelve studies [28-39] reported improvements in HbA_1c_ levels among home-based patients with T2DM following the use of digital diabetes management technologies, 7 studies reported that digital diabetes management technologies effectively improve FBG levels [28-34], and 5 studies examined the impact on PBG levels [29-33]. In conclusion, digital diabetes management technologies play a pivotal role in blood glucose management for home-based patients with T2DM and should be widely implemented in clinical practice. Ongoing focus on blood glucose monitoring is essential for maintaining stable blood sugar levels in these patients [43].

At the same time, the meta-analysis results of this study suggest that digital diabetes management technologies can reduce the BMI of home-based patients with T2DM, consistent with the findings of a systematic review by Marcolino et al [44]. However, the heterogeneity in this study is relatively high. Sensitivity analysis, conducted by excluding studies one at a time, indicated that the variability may be due to differences in intervention durations. Subgroup analysis based on intervention duration showed that when the intervention lasted less than 3 months, it effectively improved BMI. In contrast, when the intervention duration exceeded 6 months, no similar improvement was observed. Given the relatively small sample size, future large-scale, double-blind randomized controlled trials are needed to further validate the impact of digital diabetes management technologies on BMI.

With continuous innovation in sensor, microelectronics, and data analysis technologies, researchers have been actively investing in research and development. Meanwhile, the market’s strong demand for convenient and efficient blood glucose monitoring has spurred the rapid development of continuous glucose monitoring (CGM) technology. Medical staff assist patients in accessing blood glucose data via supporting mobile apps to formulate treatment plans. CGM technology offers continuous, dynamic blood glucose data, enabling a comprehensive grasp of blood glucose fluctuations, allowing timely detection of asymptomatic hypoglycemia and hyperglycemia, and contributing to optimized treatment plans and prevention of diabetes complications.

Nonetheless, CGM technology has limitations. Its equipment is costly, making long-term use unaffordable for some patients. In addition, frequent blood glucose checks may cause psychological stress in some, negatively affecting blood glucose control. Therefore, SMBG continues to play an indispensable role in diabetes management. SMBG offers patients immediate blood glucose readings at specific time points, enabling prompt adjustments to diet, exercise, or insulin dosage to maintain glucose levels within a safe range and prevent hypoglycemia or hyperglycemia. Furthermore, compared to CGM, SMBG devices are simpler, more affordable, and thus more accessible to a broader patient population, especially those with limited financial resources or relatively stable glucose fluctuations. SMBG also promotes active patient participation, increasing their awareness of disease management and enabling them to better understand the impact of daily factors on blood glucose levels. This process fosters improved lifestyle adjustments, enhances confidence in glucose control, and strengthens compliance with self-management regimens.

Among the 12 included randomized controlled trials, only 2 studies evaluated the impact of SMBG frequency on blood glucose levels [28,36], so a meta-analysis was not conducted. Both studies demonstrated that more frequent home blood glucose monitoring improved blood glucose control in home-based patients with T2DM. We observed that the frequency of SMBG in the intervention group was significantly higher than in the control group, and the digital diabetes management technology notably enhanced blood glucose control. Tomah et al [45] reported that higher SMBG frequency correlated with greater improvements in HbA_1c_, suggesting that the frequency of measurements may directly influence clinically relevant outcomes, with 4 to 8 measurements per day being the most effective. Similarly, Mannucci et al [46] found that SMBG was more effective when a structured monitoring plan was implemented, and HCPs used the collected data. None of the 12 randomized controlled trials included in this study considered the frequency of contact with HCPs as an outcome measure to assess improvements in blood glucose levels. As a result, a meta-analysis could not be conducted. However, an analysis of the intervention strategies in these studies revealed that the frequency of contact with HCPs was higher in the intervention group than in the control group. In addition, a systematic review found that SMBG without support from HCPs did not yield significant clinical benefits [47]. This lack of benefit may be attributed to treatment inertia, where poor adherence to lifestyle changes and prescribed medications is common among patients with T2DM [48,49]. When feedback from HCPs is minimal, patients may feel anxious and uncertain when faced with poor SMBG control. In conclusion, digital diabetes management technologies, along with SMBG supported by HCPs, can significantly improve blood glucose control in home-based patients with T2DM.

This study advocates for the promotion of digital diabetes management technologies based on digital SMBG for home-based patients with T2DM. Such technologies can help eliminate transcription errors associated with manual data entry and offer increased convenience for patients, especially older adult individuals with T2DM, who may struggle with inputting blood glucose values due to poor vision or limited dexterity. This technology provides an age-friendly solution for blood glucose management. In addition, for older adult patients who live alone or have limited mobility, HCPs can remotely monitor blood glucose data, offer timely care, and quickly detect and address hypoglycemia or hyperglycemia. This improves the continuity and responsiveness of diabetes management. Furthermore, the use of digital devices in clinical practice can reduce the workload of clinical nurses and enhance overall work efficiency. Thus, it is recommended that this technology be widely implemented in both home and clinical settings to improve the safety and efficiency of diabetes management.

### Comparison to Previous Work

The results of this meta-analysis align with those of previous studies on the effectiveness of digital diabetes management technology for improving blood glucose levels in home-based patients with T2DM. However, in many previous studies, SMBG devices required manual data entry for glucose measurements, whereas this study focuses on digital SMBG technologies capable of automatic data uploading [50-56]. To date, no other meta-analysis has specifically evaluated the application of digital diabetes management technology based on automatic SMBG for home-based patients with T2DM.

In addition, the results of this study revealed that digital diabetes management technology can significantly reduce BMI levels in home-based patients with T2DM, consistent with the findings of a systematic review by Marcolino et al [44]. However, several other systematic reviews suggest that digital diabetes management technology has no significant effect on BMI improvement [57-59]. This discrepancy highlights the need for future high-quality, large-sample studies to further investigate the impact of digital diabetes management technology on BMI. Such studies should explore potential differences in results due to variations in patient populations, intervention measures, and follow-up durations. Moreover, standardizing research methodologies and evaluation criteria will enhance the comparability and reliability of findings, providing a stronger evidence base for determining whether this technology can effectively improve BMI in patients with T2DM.

### Strengths and Limitations

This systematic review and meta-analysis has several notable strengths. First, to the best of our knowledge, it is the first meta-analysis to evaluate digital diabetes management techniques based on digital SMBG for improving blood glucose levels and BMI in home-based patients with T2DM. Second, a comprehensive search of multiple databases was conducted, with strict adherence to established methodological tools to ensure the reliability and transparency of the research process. Third, this study included a detailed subgroup analysis of BMI outcomes, confirming that the duration of intervention significantly influences the effectiveness of digital diabetes management technologies in home-based patients with T2DM. Finally, by analyzing multiple outcome indicators, this review provides a more comprehensive understanding of the role of digital health in diabetes management, offering valuable references for clinical practice.

We also acknowledge that this meta-analysis has some limitations. First, confined to Chinese and English literature, it excluded non–English and Chinese materials, failing to reflect global research. Future reviews should cover more languages. Second, most selected studies lack blinding details, risking measurement biases. Future research must focus on blinded outcome evaluation to minimize performance biases. Third, the HbA_1c_ funnel plot asymmetry indicates publication bias. Future studies should use multicenter, large-sample, randomized controlled trials for reliable results. Fourth, done over a year ago, the study may miss recent research due to the field’s rapid development, and lacking timeliness. Future research needs constant updates. Fifth, 8 (67%) of the 12 studies were from China, with regional limitations as China’s medical resources and telemedicine acceptance differ from elsewhere. Future studies should include more diverse regions to boost conclusion generality.

### Future Directions

Overall, future digital health research can fill the existing gaps and improve the approach in 5 key areas. First, for the diabetes management technology based on SMBG, continuous exploration should be carried out on its advantages in blood glucose control. In particular, the impact of SMBG supported by HCPs on blood glucose should be assessed to tap more potential. Second, the intervention measures should be elaborated in detail so as to achieve the maximum degree of reproducibility and universality in different clinical contexts. Third, a comprehensive evaluation system will be constructed, covering aspects such as cost-effectiveness, patient satisfaction, and technology availability. This system is used to measure the value of intervention measures for the sustainable development of digital health in diabetes management. Fourth, researchers should be encouraged to study the differences in the efficacy of different population subgroups or provide patient-level data, enabling future reviews to identify the patient characteristics related to treatment outcomes. Fifth, researchers should actively collect and report patients’ views on the intervention measures to improve the design basis.

### Conclusion

This meta-analysis demonstrates that digital diabetes management technologies can effectively regulate blood glucose levels, improve glucose metabolism, and reduce BMI in patients with T2DM. However, due to the variation in intervention strategies, frequencies, and content across the included studies, future research should prioritize multicenter, large-sample, high-quality randomized controlled trials to refine and standardize these interventions. Notably, effective digital health interventions share a common characteristic: frequent patient engagement in SMBG, supported by HCPs who provide timely and personalized guidance.

## Appendix

Multimedia Appendix 1 Search strategy.

Multimedia Appendix 2 Characteristics of the 12 included studies.

Multimedia Appendix 3 PRISMA (Preferred Reporting Items for Systematic Reviews and Meta-Analyses) Checklist.

## Abbreviations

CGM: continuous glucose monitoring
FBG: fasting blood glucose
HbA_1c_: hemoglobin A_1c_
HCP: health care professional
MD: mean difference
PBG: postprandial blood glucose
PICOS: populations, interventions, comparisons, outcomes, and study design
PRISMA: Preferred Reporting Items for Systematic Reviews and Meta-Analyses
SMBG: self-monitoring of blood glucose
T2DM: type 2 diabetes mellitus

## Data Availability

All data generated or analyzed during this study are included in this published paper and its supplementary information files.
