# Supplementary material for: Effectiveness of Digital Diabetes Management Technology on Blood Glucose in Patients With Type 2 Diabetes at Home: Systematic Review and Meta-Analysis
Source: J Med Internet Res. 2025 Mar 3;27:e66441. doi: 10.2196/66441 (PMC11914849; doi:10.2196/66441)
Supplement: Multimedia Appendix 1 [file jmir_v27i1e66441_app1.docx]

Appendix 1

| Pubmed | | |
| --- | --- | --- |
| 23/11/2023 | | |
| #1 | Diabetes Mellitus, Type 2[MeSH Terms] | 174,986 |
| #2 | (((((((((((((((((((((((((((((Diabetes Mellitus, Noninsulin-Dependent[Title/Abstract]) OR (Diabetes Mellitus, Ketosis-Resistant[Title/Abstract])) OR (Diabetes Mellitus, Ketosis Resistant[Title/Abstract])) OR (Diabetes Mellitus, Non Insulin Dependent[Title/Abstract])) OR (Diabetes Mellitus, Non-Insulin-Dependent[Title/Abstract])) OR (Non-Insulin-Dependent Diabetes Mellitus[Title/Abstract])) OR (Diabetes Mellitus, Stable[Title/Abstract])) OR (Stable Diabetes Mellitus[Title/Abstract])) OR (Diabetes Mellitus, Type II[Title/Abstract])) OR (NIDDM[Title/Abstract])) OR (Diabetes Mellitus, Noninsulin Dependent[Title/Abstract])) OR (Diabetes Mellitus, Maturity-Onset[Title/Abstract])) OR (Diabetes Mellitus, Maturity Onset[Title/Abstract])) OR (Maturity-Onset Diabetes Mellitus[Title/Abstract])) OR (Maturity Onset Diabetes Mellitus[Title/Abstract])) OR (Maturity Onset Diabetes Mellitus[Title/Abstract])) OR (Diabetes Mellitus, Slow-Onset[Title/Abstract])) OR (Diabetes Mellitus, Slow Onset[Title/Abstract])) OR (Slow-Onset Diabetes Mellitus[Title/Abstract])) OR (Type 2 Diabetes Mellitus[Title/Abstract])) OR (Noninsulin-Dependent Diabetes Mellitus[Title/Abstract])) OR (Noninsulin Dependent Diabetes Mellitus[Title/Abstract])) OR (Maturity-Onset Diabetes[Title/Abstract])) OR (Diabetes, Maturity-Onset[Title/Abstract])) OR (Maturity Onset Diabetes[Title/Abstract])) OR (Type 2 Diabetes[Title/Abstract])) OR (Diabetes, Type 2[Title/Abstract])) OR (Diabetes Mellitus, Adult-Onset[Title/Abstract])) OR (Adult-Onset Diabetes Mellitus[Title/Abstract])) OR (Diabetes Mellitus, Adult Onset[Title/Abstract]) | 185,358 |
| #3 | #1 OR #2 | 238,349 |
| #4 | Internet[MeSH Terms] | 1,086 |
| #5 | ((((((((((((((Telemedicine[Title/Abstract]) OR (Virtual Medicine[Title/Abstract])) OR (Mobile Health[Title/Abstract])) OR (mHealth[Title/Abstract])) OR (Telehealth[Title/Abstract])) OR (eHealth[Title/Abstract])) OR (Telemetry[Title/Abstract])) OR (telemonitoring[Title/Abstract])) OR (Internet of things[Title/Abstract])) OR (Telerehabilitation[Title/Abstract])) OR (Remote Rehabilitation[Title/Abstract])) OR (Remote Rehabilitations[Title/Abstract])) OR (Virtual Rehabilitation[Title/Abstract])) OR (Virtual Rehabilitations[Title/Abstract])) OR (Monitoring, Home Blood Glucose[Title/Abstract]) | 140,488 |
| #6 | #4 OR #5 | 140,520 |
| #7 | #3 AND #6 | 1,830 |
| #8 | randomized controlled trial | 809,976 |
| #9 | #7 AND #8 | 502 |
| Embase | | |
| 2/11/2023 | | |
| #1 | ('diabetes'/exp OR diabetes) AND mellitus, AND type AND ('2'/exp OR 2) | 430,819 |
| #2 | 'Diabetes Mellitus, Noninsulin-Dependent':ab,ti OR 'Diabetes Mellitus, Ketosis-Resistant':ab,ti OR 'Diabetes Mellitus, Ketosis Resistant':ab,ti OR 'Diabetes Mellitus, Non Insulin Dependent':ab,ti OR 'Diabetes Mellitus, Non-Insulin-Dependent':ab,ti OR 'Non-Insulin-Dependent Diabetes Mellitus':ab,ti OR 'Diabetes Mellitus, Stable':ab,ti OR 'Stable Diabetes Mellitus':ab,ti OR 'Diabetes Mellitus, Type II':ab,ti OR 'NIDDM':ab,ti OR 'Diabetes Mellitus, Noninsulin Dependent':ab,ti OR 'Diabetes Mellitus, Maturity-Onset':ab,ti OR 'Diabetes Mellitus, Maturity Onset':ab,ti OR 'Maturity-Onset Diabetes Mellitus':ab,ti OR 'Maturity Onset Diabetes Mellitus':ab,ti OR 'MODY':ab,ti OR 'Diabetes Mellitus, Slow-Onset':ab,ti OR 'Diabetes Mellitus, Slow Onset':ab,ti OR 'Slow-Onset Diabetes Mellitus':ab,ti OR 'Type 2 Diabetes Mellitus':ab,ti OR 'Noninsulin-Dependent Diabetes Mellitus':ab,ti OR 'Noninsulin Dependent Diabetes Mellitus':ab,ti OR 'Maturity-Onset Diabetes':ab,ti OR 'Diabetes, Maturity-Onset':ab,ti OR 'Maturity Onset Diabetes':ab,ti OR 'Type 2 Diabetes':ab,ti OR 'Diabetes, Type 2':ab,ti OR 'Diabetes Mellitus, Adult-Onset':ab,ti OR 'Adult-Onset Diabetes Mellitus':ab,ti | 275,230 |
| #3 | #1 OR #2 | 461,530 |
| #4 | 'Telemedicine':ab,ti OR 'Virtual Medicine':ab,ti OR 'Mobile Health':ab,ti OR 'mHealth':ab,ti OR 'Telehealth':ab,ti OR 'eHealth':ab,ti OR 'Telemetry':ab,ti OR 'telemonitoring ':ab,ti OR 'Internet of things':ab,ti OR 'Internet':ab,ti OR 'World Wide Web':ab,ti OR 'CyberSpace':ab,ti OR 'Telerehabilitation':ab,ti OR 'Remote Rehabilitation':ab,ti OR 'Remote Rehabilitations':ab,ti OR 'Virtual Rehabilitation':ab,ti OR 'Virtual Rehabilitations':ab,ti OR 'Monitoring, Home Blood Glucose':ab,ti | 168,612 |
| #5 | #3 AND #4 | 3039 |
| #6 | randomized AND controlled AND trial | 1,148,319 |
| #7 | #5 AND #6 | 450 |
| Web of Science Collection | | |
| 2/11/2023 | | |
| #1 | TS=（Diabetes Mellitus, Type 2 OR Diabetes Mellitus, Noninsulin-Dependent OR Diabetes Mellitus, Ketosis-Resistant OR Diabetes Mellitus, Ketosis Resistant OR Diabetes Mellitus, Non Insulin Dependent OR Diabetes Mellitus, Non-Insulin-Dependent OR Non-Insulin-Dependent Diabetes Mellitus OR Diabetes Mellitus, Stable OR Stable Diabetes Mellitus OR Diabetes Mellitus, Type II OR NIDDM OR Diabetes Mellitus, Noninsulin Dependent OR Diabetes Mellitus, Maturity-Onset OR Diabetes Mellitus, Maturity Onset OR Maturity-Onset Diabetes Mellitus OR Maturity Onset Diabetes Mellitus OR MODY OR Diabetes Mellitus, Slow-Onset OR Diabetes Mellitus, Slow Onset OR Slow-Onset Diabetes Mellitus OR Type 2 Diabetes Mellitus OR Noninsulin-Dependent Diabetes Mellitus OR Noninsulin Dependent Diabetes Mellitus OR Maturity-Onset Diabetes OR Diabetes, Maturity-Onset OR Maturity Onset Diabetes OR Type 2 Diabetes OR Diabetes, Type 2 OR Diabetes Mellitus, Adult-Onset OR Adult-Onset Diabetes Mellitus ） | 286,343 |
| #2 | TS=( Telemedicine OR Virtual Medicine OR Mobile Health OR mHealth OR Telehealth OR eHealth OR Telemetry OR telemonitoring OR Internet of things OR Internet OR World Wide Web OR CyberSpace OR Telerehabilitation OR Remote Rehabilitation OR Remote Rehabilitations OR Virtual Rehabilitation OR Virtual Rehabilitations OR Monitoring, Home Blood Glucose ) | 576,917 |
| #3 | TS=( randomized controlled trial) | 532,471 |
| #4 | #1 AND #2 AND #3 | 444 |
| CENTRAL (The Cochrane Central Registered of Controlled Trials) | | |
| 3/11/2023 | | |
| #1 | Diabetes Mellitus, Type 2 | 6,9502 |
| #2 | (Diabetes Mellitus, Noninsulin-Dependent):ab,ti,kw OR (Diabetes Mellitus, Ketosis-Resistant):ab,ti,kw OR(Diabetes Mellitus, Ketosis Resistant):ab,ti,kw OR(Diabetes Mellitus, Non Insulin Dependent):ab,ti,kw OR(Diabetes Mellitus, Non-Insulin-Dependent):ab,ti,kw OR(Non-Insulin-Dependent Diabetes Mellitus):ab,ti,kw OR(Diabetes Mellitus, Stable):ab,ti,kw OR(Stable Diabetes Mellitus):ab,ti,kw OR(Diabetes Mellitus, Type II):ab,ti,kw OR(NIDDM):ab,ti,kw OR(Diabetes Mellitus, Noninsulin Dependent):ab,ti,kw OR(Diabetes Mellitus, Maturity-Onset):ab,ti,kw OR(Diabetes Mellitus, Maturity Onset):ab,ti,kw OR(Maturity-Onset Diabetes Mellitus):ab,ti,kw OR(Maturity Onset Diabetes Mellitus):ab,ti,kw OR(MODY):ab,ti,kw OR(Diabetes Mellitus, Slow-Onset):ab,ti,kw OR(Diabetes Mellitus, Slow Onset):ab,ti,kw OR(Slow-Onset Diabetes Mellitus):ab,ti,kw OR(Type 2 Diabetes Mellitus):ab,ti,kw OR(Noninsulin-Dependent Diabetes Mellitus):ab,ti,kw OR(Noninsulin Dependent Diabetes Mellitus):ab,ti,kw OR(Maturity-Onset Diabetes):ab,ti,kw OR(Diabetes, Maturity-Onset):ab,ti,kw OR(Maturity Onset Diabetes):ab,ti,kw OR(Type 2 Diabetes):ab,ti,kw OR(Diabetes, Type 2):ab,ti,kw OR(Diabetes Mellitus, Adult-Onset):ab,ti,kw OR(Adult-Onset Diabetes Mellitus):ab,ti,kw | 6,3201 |
| #3 | #1 OR #2 | 7,8611 |
| #2 | (Telemedicine):ab,ti,kw OR (Virtual Medicine):ab,ti,kw OR (Mobile Health):ab,ti,kw OR (mHealth):ab,ti,kw OR (Telehealth):ab,ti,kw OR (eHealth):ab,ti,kw OR (Telemetry):ab,ti,kw OR (telemonitoring ):ab,ti,kw OR (Internet of things):ab,ti,kw OR (Internet):ab,ti,kw OR (World Wide Web):ab,ti,kw OR (CyberSpace):ab,ti,kw OR (Telerehabilitation):ab,ti,kw OR (Remote Rehabilitation):ab,ti,kw OR (Remote Rehabilitations):ab,ti,kw OR (Virtual Rehabilitation):ab,ti,kw OR (Virtual Rehabilitations):ab,ti,kw OR (Monitoring, Home Blood Glucose):ab,ti,kw | 3,5980 |
| #3 | randomized controlled trial | 1,042,216 |
| #4 | #1 AND #2 AND #3 | 204 |
| China National Knowledge Infrastructure (CNKI) | | |
| 1/11/2023 | | |
| #1 | 主题 （糖尿病，2型 or 糖尿病2型 or 2型糖尿病 or 第二型糖尿病 or 成熟型糖尿病 or 成人发病型糖尿病 or 非胰岛素依赖型糖尿病 or 二型糖尿病 or 非胰岛素依赖性糖尿病） | 113,573 |
| #2 | 主题（物联网 or 远程医疗 or 互联网 or 延续性护理 or 院外护理 or 信息化管理） | 790,723 |
| #3 | 主题（血糖 or 自我管理 or 生活质量 or 治疗满意度 ） | 1,017,015 |
| #4 | 摘要（随机对照试验 or 随机对照实验 or 随机对照研究 or RCT or 随机对照 or 随机 ） | 2,427,778 |
| #5 | #1 AND #2 AND #3 AND #4 | 1934 |
| WANFANG DATA | | |
| 1/11/2023 | | |
| #1 | 主题（糖尿病，2型 or 糖尿病2型 or 2型糖尿病 or 第二型糖尿病 or 成熟型糖尿病 or 成人发病型糖尿病 or 非胰岛素依赖型糖尿病 or 二型糖尿病 or 非胰岛素依赖性糖尿病） | 744720 |
| #2 | 主题（物联网 or 远程医疗 or 互联网 or 延续性护理 or 院外护理 or 信息化管理） | 161172 |
| #3 | 主题（血糖 or 自我管理 or 生活质量 or 治疗满意度） | 2491227 |
| #4 | 主题（随机对照试验 or 随机对照实验 or 随机对照研究 or RCT or 随机对照 or 随机） | 3260393 |
| #5 | #1 AND #2 AND #3 AND #4 | 761 |
| Chinese Biomedical Literature Database (CBM) | | |
| 23/11/2023 | | |
| #1 | "糖尿病, 2型"[不加权:扩展] | 1,,030,53 |
| #2 | "糖尿病2型"[常用字段:智能] OR "2型糖尿病"[常用字段:智能] OR "第二型糖尿病"[常用字段:智能] OR "成熟型糖尿病"[常用字段:智能] OR "成人发病型糖尿病"[常用字段:智能] OR "非胰岛素依赖型糖尿病"[常用字段:智能] OR "二型糖尿病"[常用字段:智能] OR "非胰岛素依赖性糖尿病"[常用字段:智能] | 1,362,38 |
| #3 | #1 OR #2 | 1,362,38 |
| #4 | "物联网"[不加权:扩展] | 1196 |
| #5 | "远程医疗"[常用字段:智能] OR "互联网"[常用字段:智能] OR "延续性护理"[常用字段:智能] OR "院外护理"[常用字段:智能] OR "信息化管理"[常用字段:智能] | 3,9733 |
| #6 | #4 OR #5 | 4,0710 |
| #7 | "随机对照试验"[不加权:扩展] | 1,965，14 |
| #8 | "随机对照实验"[常用字段:智能] OR "随机对照研究"[常用字段:智能] OR "RCT"[常用字段:智能] OR "随机对照"[常用字段:智能] OR "随机"[常用字段:智能] | 1,918,508 |
| #9 | #7 OR #8 | 1,918,723 |
| #10 | #3 AND #6 AND #9 | 882 |

**Search terms list**

| **Category** | **Search terms** |
| --- | --- |
| Diabetes Mellitus, Type 2 | Diabetes Mellitus, Noninsulin-Dependent or Diabetes Mellitus, Ketosis-Resistant or Diabetes Mellitus, Ketosis Resistant or Diabetes Mellitus, Non Insulin Dependent  or Diabetes Mellitus, Non-Insulin-Dependent or Non-Insulin-Dependent Diabetes or or Mellitus or Diabetes Mellitus, Stable or Stable Diabetes Mellitus or Diabetes Mellitus, Type II or NIDDM or Diabetes Mellitus, Noninsulin Dependent or Diabetes Mellitus, Maturity-Onset or Diabetes Mellitus, Maturity Onset or Maturity-Onset Diabetes Mellitus or Maturity Onset Diabetes Mellitus or MODY or Diabetes Mellitus, Slow-Onset or Diabetes Mellitus, Slow Onset or Slow-Onset Diabetes Mellitus or Type 2 Diabetes Mellitus or Noninsulin-Dependent Diabetes Mellitus or Noninsulin Dependent Diabetes Mellitus or Maturity-Onset Diabetes or Diabetes, Maturity-Onset or Maturity Onset Diabetes or Type 2 Diabetes or Diabetes, Type 2 or Diabetes Mellitus, Adult-Onset or Adult-Onset Diabetes Mellitus |
| Internet | Telemedicine or Virtual Medicine or Mobile Health or mHealth or Telehealth or eHealth or Telemetry or telemonitoring or Internet of things or Telerehabilitation or Remote Rehabilitation or Remote Rehabilitations or Virtual Rehabilitation or Virtual Rehabilitations or Monitoring, Home Blood Glucose |
| randomized controlled tria |  |
