# Supplementary material for: Effectiveness of Digital Diabetes Management Technology on Blood Glucose in Patients With Type 2 Diabetes at Home: Systematic Review and Meta-Analysis
Source: J Med Internet Res. 2025 Mar 3;27:e66441. doi: 10.2196/66441 (PMC11914849; doi:10.2196/66441)
Supplement: Multimedia Appendix 2 [file jmir_v27i1e66441_app2.docx]

**Multimedia Appendix 2.** Characteristics of the 12 included studies.

| Author (Reference) | Year | Country | sample size | Equipment used by the intervention | Intervening measure | Control measure | Duration | Outcomes |
| --- | --- | --- | --- | --- | --- | --- | --- | --- |
| Antonio Nicolucci [1] | 2015 | Italy | I^a^:114; C^b^:135 | Blood glucose meter (model G31; Fora Care), Home telehealth | Blood glucose meter connects to a hub via Bluetooth, blood glucose data is transmitted and stored in a centralized system, and General practitioners can provide remote guidance through the home telemedicine system | Continue to be routinely followed up by General practitioners | 12 months | HbA_1C_^c^ |
| Chen-Yu Han [2] | 2023 | China | I:115; C:65 | Glucose meter (SINOMEDISITE), mobile app (Huayi Glucose Butler) | Patients transmit blood glucose monitoring data to the telemedicine system in real time via Bluetooth and receive remote guidance | Routine care, 1 follow-up visit per month | 6 months | HbA_1C_;FBG^d^;BMI |
| Chenglin Sun [3] | 2019 | China | I:44; C:47 | mHealth management app | Patients use the mHealth management application, and the blood glucose data monitored by the glucose meter is automatically transmitted to the healthcare server via Bluetooth to receive remote guidance | Receive a free blood glucose meter and follow up with a routine clinic appointment | 6 months | HbA_1C_; FBG;PBG^e^; BMI |
| Xiling Zhao [4] | 2021 | China | I:50; C:50 | Blood glucose information management system (iGMS), intelligent blood glucose meter, sugar butler APP | Patients in the intervention group completed home blood glucose level monitoring using personal smart glucose meters and the Glucose Manager App | Routine telephone follow-up | 3 months | HbA1C;FBG;PBG;BMI |
| Mengna Guo [5] | 2021 | China | I:32; C:32 | mHealth management based on themHealth management model that consisted of the network platform, an implantable glucose sensor, a mobile app and General Practitioner support | Blood glucose values collected by implantable glucose sensors can be automatically transmitted to a mobile app. Through this app, physicians can receive real-time information and develop a plan that can be automatically transmitted to the patient | Routine care, weekly telephone follow-ups, and urging patients to attend outpatient clinics for review on time | 1 month | HbA1C;FBG;PBG;BMI |
| Sarah H. Wild [6] | 2016 | England | I:146; C:139 | a validated glucose meter, modem | Blood glucose monitors for patients in the intervention group used Bluetooth wireless technology to transmit readings to a remote secure server via a modem provided by the nurse | Routine care, reviewed at least once a year | 9 months | HbA1C |
| Wenwen Yin [7] | 2022 | China | I:52; C:47 | hospital telemedicine app, glucometer was connected to the patient’s mobile phone via Bluetooth | The glucose meter connects to the patient's cell phone via Bluetooth, which then automatically transfers the data to the app | Weekly follow-up by phone and every 2 weeks through regular clinic appointments | 6 months | HbA1C;FBG;PBG;BMI |
| Yuli Hu [8] | 2020 | India | I:72; C:70 | EZ-6 Smart Blood Glucose Meter (sinomedisite) | The blood glucose data measured by the smart glucose meter is automatically transmitted to the telemedicine system via the Internet | Received routine outpatient care once a month from endocrinologists and nurses | 6 months | HbA1C |
| Yakun Feng [9] | 2016 | China | I:97; C:89 | Myglucohealth (Infopia) blood glucose meter, Diabetes U-Healthcare | Transmit blood glucose data using a glucose meter with data transmission function and obtain comprehensive diabetes U-Healthcare management services | Traditional outpatient visits | 6 months | HbA1C;FBG;PBG;BMI |
| Yan Wang [10] | 2017 | China | I:39; C:37 | Intelligent blood glucose monitor (GLUPAD), blood glucose information management system | Monitor blood glucose through the smart glucose meter. It has real-time connection to the Internet and is synchronized to the blood glucose information management system | Glucose monitoring using a portable glucose meter and completing a glucose monitoring logbook | 6 months | HbA1C |
| Zhiwei Lu [11] | 2021 | China | I:60; C:59 | Blood glucose meter (ACCU-CHECK performa Connect) | Patients can transmit data from their blood glucose meters to the service platform via Bluetooth | Outpatient visits every 2 weeks starting 14 days after hospital discharge | 6 months | HbA1C;FBG; |
| Issac Sachmechi [12] | 2023 | New York | I:39; C:39 | Vivovitals platform, Continuous Glucose Monitoring equipment | Data from the glucose meter is automatically transferred to the Vivovitals app | Routine clinical care, 12-week outpatient visits | 3 months | HbA1C |

^a^I: intervention.

^b^C: control.

^c^HbA1C: Hemoglobin A1c.

^d^FBG: fasting blood sugar.

^e^PBG: postprandial blood glucose.

**References**

1. Nicolucci A, Cercone S, ChiriattiA, MuscasF, Gensini G. A randomized trial on home telemonitoring for the management of metabolic and cardiovascular risk inpatients with Type 2 diabetes. Diabetes Technol Ther. 2015;17(8):563-570. [doi: [10.1089/dia.2014.0355](http://dx.doi.org/10.1089/dia.2014.0355)] [Medline: [26154338](http://www.ncbi.nlm.nih.gov/entrez/query.fcgi?cmd=Retrieve&db=PubMed&list_uids=26154338&dopt=Abstract)]
2. Han CY, Zhang J, Ye XM, Lu JP, Jin HY, Xu WW, et al. Telemedicine-assisted structured self-monitoring of blood glucose in management ofT2DM results of a randomized clinical trial. BMC Med Inform Decis Mak. 2023;23(1):182. [FREE Full text] [doi: 10.1186/s12911-023-02283-4] [Medline: 37710226]
3. Sun C, Sun L, Xi S, Zhang H, Wang H, Feng Y, et al. Mobile phone-based telemedicine practice in older Chinese patients with type 2 diabetes mellitus: randomized controlled trial. JMIR Mhealth Uhealth. 2019;7(1):e10664. [FREE Full text] [doi: 10.2196/10664] [Medline: 30609983]
4. Zhao XL. Impact of blood glucose information management system on body composition, blood glucose, blood lipids and self-management ability of patients with type 2 diabetes mellitus. J Chronic Dis. 2021;22(06):871-874. [doi:10.16440/j.cnki.1674-8166.2021.06.014]
5. Guo M, Meng F, Guo Q, Bai T, Hong Y, Song F, et al. Effectiveness of mHealth management with an implantable glucose sensor and a mobile application among Chinese adults with type 2 diabetes. J Telemed Telecare. 2023;29(8):632-640. [doi: [10.1177/1357633X211020261](http://dx.doi.org/10.1177/1357633X211020261)] [Medline: [34152238](http://www.ncbi.nlm.nih.gov/entrez/query.fcgi?cmd=Retrieve&db=PubMed&list_uids=34152238&dopt=Abstract)]
6. Wild SH, Hanley J, Lewis SC, McKnight JA, McCloughan LB, Padfield PL, et al. Supported telemonitoring and glycemic control in people with type 2 diabetes: the telescot diabetes pragmatic multicenter randomized controlled trial. PLoS Med. 2016;13(7):e1002098. [FREE Full text] [doi: 10.1371/journal.pmed.1002098] [Medline: 27458809]
7. Yin W, Liu Y, Hu H, Sun J, Liu Y, Wang Z. Telemedicine management oftype 2 diabetes mellitus in obese and overweight young and middle-aged patients during COVID-19 outbreak: a single-center, prospective, randomized control study. PLoS One. 2022;17(9):e0275251. [FREE Full text] [doi: 10.1371/journal.pone.0275251] [Medline: 36174028]
8. HuY, Wen X, NiL, Wang F, Hu S, Fang F. Effects of telemedicine intervention on the management of diabetic complications in type 2 diabetes. Int J Diabetes Dev Ctries. 2020;41(2):322-328. [FREE Full text] [doi: 10.1007/s13410-020-00893-6]
9. Ya-Kun E, Yu-JiaL, Xiao-Kun G. The application of telemedicine in the integrated management of type 2 diabetes mellitus. Chin J Diabetes. 2016;24(05):439-442. [doi: 10.3969/j.issn.1006-6187.2016.05.013]
10. Yan W, Shanshan Y, Jiaping N, Xiaojing L. Application effect of blood glucose information management system on the follow-up among patients with type 2 diabetes. Chin Nurs Manage. 2017;17(09):1245-1248. [doi:10.3969/j.issn.1672-1756.2017.09.021]
11. LuZ, Li Y, HeY, ZhaiY, Wu J, Wang J,et al. Internet-based medication management services improve glycated hemoglobin levels inpatients with type 2 diabetes. Telemed J E Health. 2021;27(6):686-693. [doi: [10.1089/tmj.2020.0123](http://dx.doi.org/10.1089/tmj.2020.0123)] [Medline: [32907521](http://www.ncbi.nlm.nih.gov/entrez/query.fcgi?cmd=Retrieve&db=PubMed&list_uids=32907521&dopt=Abstract)]
12. Sachmechi I, Salam S,AminiM, Khan R, Spitznogle A, Belen T. Frequent monitoring of blood glucose levels via a remote patient monitoring system helps improve glycemic control. Endocr Pract. 2023;29(6):441-447. [doi:10.1016/j.eprac.2023.03.270] [Medline: 36965657]
